# 

**Published:** 1882-09

**Authors:** 


### CONTENTS.

### *MISCELLANEOUS.*

PAGE

The American Institute, Session of 1882, . . . . . . . 361
The Recognition. Ad. Lippe, M. D., Philadelphia, . . . . . 368
The Second Annual Meeting of the International Hahnemannian Association, . . . . . . . . . . . . . 372
Roll of Members of the International Hahnemannian Association, . 377
Microscopic Falsehoods, . . . . . . . . . . . 379

### *CLINICAL BUREAU.*

Carcinoma. L. B. Wells, M. D., Utica, New York, . . . . . 379

Infantile Convulsions. O. P. Baer, M. D., Richmond, Indiana, . . 381

Case of Puerperal Fever, followed by Phlegmasia Alba Dolens. E. W. Berridge, M. D., London, . . . . . . . . . 388

Cases from Practice, with Remarks. C. Pearson, M. D., Washington, D. C., . . . . . . . . . . . . . 398

Cases from Practice. J. B. Gregg Custis, M. D., Washington, D. C., . 401

### *BOOK NOTICES.*

American Medicinal Plants. Bœricke & Tafel, . . . . . . 404
A Hand-book on the Diseases of the Heart, and Their Homœopathetic Treatment. Duncan Brothers, . . . . . . . . 404
Cough Symptoms, . . . . . . . . . . . . 25–28

